# Predicting histological grade in invasive ductal carcinoma of the breast: a radiomics-based machine learning model using DCE-MRI

**DOI:** 10.3389/fonc.2025.1593075

**Published:** 2026-01-23

**Authors:** Ziwen Wang, Chenglin Bai, Naiyou Zhang, Zhipeng Han, Haiming Dong, Shanzheng Liu, Jingjing Meng, Chengjun Zhang

**Affiliations:** 1 Radiology Department of Chaoyang Central Hospital Affiliated to China Medical University, Chaoyang, Liaoning, China; 2 Breast Surgery Department of Chaoyang Central Hospital Affiliated to China Medical University, Chaoyang, Liaoning, China; 3 Pathology Department of Chaoyang Central Hospital Affiliated to China Medical University, Chaoyang, Liaoning, China

**Keywords:** breast cancer, dynamic contrast-enhanced magnetic resonance imaging, histological grade, machine learning, radiomics

## Abstract

**Objectives:**

To investigate the feasibility analysis of predicting the pathological differentiation grade of breast invasive ductal carcinoma based on DCE-MRI imaging histology.

**Methodology:**

198 patients with breast invasive ductal carcinoma who underwent preoperative enhanced MRI were retrospectively collected from January 2019 to October 2024.According to Nottingham histologic grading, 108 cases were divided into a high-grade group and 90 cases into an intermediate-low-grade group, which were randomly divided into 148 cases of the training group and 50 cases of the validation group according to a 3:1 ratio. The 3D slicer software was applied to extract the image histological features of the region of interest, and five models, namely, decision tree, Gaussian plain Bayes, logistic regression, random forest, and AdaBoost, were constructed by filtering the features with intragroup correlation coefficients and the minimum absolute contraction and selection operators. Compare the area under the work characteristic curve of subjects in the validation group and select the best model. The performance of the best model validation group was evaluated, the clinical usability of the best model was examined using decision curves, and the accuracy of the predictive model was visualized using calibration curves.

**Results:**

After rigorous stability and redundancy screening, 22 key radiomics features were selected from DCE-MRI images. Multiple machine learning models trained based on these features were evaluated for their predictive performance on the validation set. The logistic regression model achieved the highest AUC value of 0.795 (95% confidence interval: 0.664-0.927), outperforming other models such as random forest (AUC = 0.700), Gaussian naive Bayes (AUC = 0.700), AdaBoost (AUC = 0.718), and decision tree (AUC = 0.587). Consequently, the logistic regression model was ultimately selected as the optimal model.

**Conclusion:**

The DCE-MRI radiomics model based on Logistic Regression can non-invasively and effectively predict the histological grade of IDC preoperatively, offering valuable potential for supporting individualized clinical decision-making.

## 1 Background

Breast cancer is one of the most common malignant tumors among women globally and its incidence poses a significant threat to women’s health and is a major public health problem (1, 2). Infiltrating ductal carcinoma is the most common type of breast cancer, accounting for 50% to 75% of all cases (3, 4). Timely diagnosis and treatment play a crucial role in the management of breast cancer and are directly related to patient survival and quality of life. Histologic grading by pathology remains the current accepted method of definitive diagnosis in the medical community. This process not only accurately identifies the type and stage of cancer, but also has a profound impact on the development of treatment plans and prognostic assessment of invasive breast cancer, helping physicians to tailor the most effective treatment plan for their patients, which in turn improves treatment efficacy and optimizes the patient’s path to recovery (5–8). Specifically, histological differentiation grade is usually highly correlated with increased neovascularization and degree of tumor malignancy (9). Recent studies have found a significant correlation between the expression of certain molecular markers and the degree of tumor differentiation. For example, HER2-positive breast cancer usually exhibits low differentiation characteristics, while ER-positive breast cancer tends to be a highly differentiated type. It reinforces that such differences in molecular characteristics not only affect the biological behavior of tumors but also provide a basis for individualized treatment (10). Therefore, preoperative prediction of tumor histological grading has significant clinical benefits.

Studies have shown that DCE-MRI has high sensitivity and specificity in the early diagnosis of breast cancer. Compared with digital mammography and digital breast tomosynthesis, DCE-MRI is more effective in identifying early breast cancer lesions, especially in women with dense breast tissue. Studies have shown that DCE-MRI outperforms conventional imaging methods in detecting occult breast cancer and can significantly improve early detection rates (11).

While DCE-MRI provides comprehensive diagnostic information, conventional imaging assessments primarily rely on physicians’ subjective visual analysis, which struggles to quantify the deep-seated heterogeneity within tumors. Radiomics is the process of transforming imaging datasets into high-dimensional mineable features using feature extraction algorithms, followed by machine learning to construct statistical models for diagnosis, classification and prediction. Radiomics as an emerging field offers a powerful tool to address this challenge (12, 13).

Recently, studies have demonstrated the novel role of radiomics in identifying breast malignancy (7). Studies by Kang et al. (14), Yu et al. (15), and Zhang et al. (16) have shown that imaging sex histology features extracted from DCE-MRI provide important information for prognostic assessment of breast tumors, as well as a potential imaging basis for evaluating the efficacy of treatment. However, research on the pathological heterogeneity within invasive ductal carcinoma (IDC) particularly the preoperative non-invasive prediction of histological differentiation remains limited and inconclusive. While pathological grading is a critical determinant of IDC prognosis and adjuvant therapy strategies, current assessments primarily rely on postoperative pathological specimens. Therefore, developing a reliable preoperative DCE-MRI-based model for precise differentiation prediction of IDC is clinically imperative to facilitate personalized neoadjuvant therapy decisions and prognostic evaluation.

Therefore, the aim of this study was to investigate the feasibility of individual machine learning models of DCE-MR in predicting the histological grade of invasive ductal carcinoma of the breast for personalized preoperative invasive ductal carcinoma treatment.

## 2 Information and methods

This study included a retrospective dataset from an academic medical center, and this study was approved by the Ethics Committee of Chaoyang Central Hospital. The request for written informed consent was waived because it was a non-interfering retrospective study. All personal information of included patients was kept strictly confidential. This study followed the 1964 Declaration of Helsinki and its subsequent amendments.

### 2.1 Study subjects

**Figure 1:**
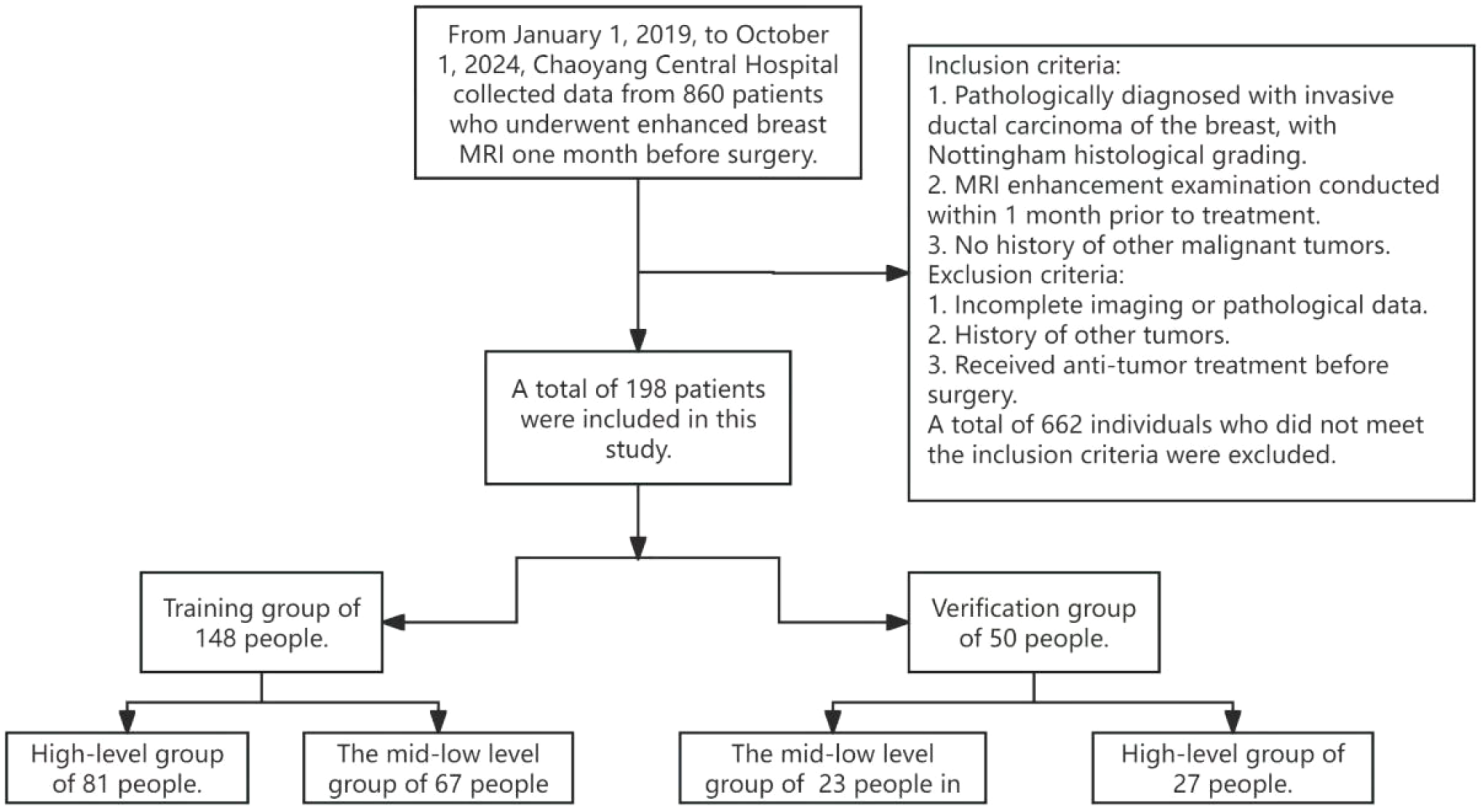
Flowchart of case screening.

Patients with pathologically confirmed invasive ductal carcinoma of the breast from January 2019 to October 2024 were retrospectively collected and numbered. Inclusion Criteria: ① Pathologic diagnosis of invasive ductal carcinoma of the breast after the first mastectomy for invasive ductal carcinoma of the breast or puncture with clear Nottingham histologic grading. ② Breast MRI enhancement examination with high quality imaging data within 1 month before here. ③ No preoperative anti-tumor therapy. Exclusion criteria: ① Incomplete imaging or pathological data; ② Suffering from other tumor history. ③ Received anti-tumor treatment before surgery. This study was approved by the Ethics Committee of Chaoyang Central Hospital (No.【2024】31). According to the Nottingham histologic grading, the pathological grading of grade III was regarded as the high-grade group, and the pathological grading of grade I and II were regarded as the middle-low-grade group. We randomly divided the subjects into a training group (n=148) and a test group (n=50) at a 3:1 ratio, as illustrated in **Figure 1**. We adopted this approximate 3:1 ratio because preliminary leave-out cross-validation experiments demonstrated that this partitioning ensures sufficient training data while delivering optimal model performance and the most stable generalization results on the test set.

### 2.2 Instruments and methods

MRI examination methods using General Electric Company SIGNA Pioneer 3.0T magnetic resonance scanner, routinely placed in the prone position, arms stretched forward to ensure that the anterior chest is exposed, the breasts are placed in the breast coil and tightly attached; dynamic enhancement scanning. Contrast agent [gadoteric acid glucosamine injection (concentration 0.2 mL/kg, injection rate 3 mL/s)] was injected simultaneously and 12 temporal phases were scanned after the scanning, and the total scanning time was about 6 min, TR = 6.24 ms, TE = 1.81 ms, FOV = 32 cm, and the layer thickness was = 2.2 mm through the consecutive spaceless acquisitions.

### 2.3 Image segmentation

MRI images were transferred in DICOM format to 3D Slicer 5.6.2 software (https://www.slicer.org). In this study, two experienced imaging physicians, who were unaware of the pathologic data, manually sketched the ROI of the lesion layer by layer on the image about 90 s after contrast injection and were guided by a senior imaging physician to confirm the formation of the volume of interest (VOI).The early phase 90 seconds after contrast injection is a key time point for dynamic scanning, based on established pharmacokinetic principles. This period typically corresponds to the peak enhancement plateau in most breast malignancies, when the contrast between tumor and normal tissue is most pronounced. Therefore, the delineation is performed around this time.

### 2.4 Feature extraction

Two physicians extracted 3D image texture features of the volume of regions of interest using the open-source Pyradiomics package in 3D Slicer and 1316 features were extracted for each lesion, including 14 shape features, 252 first-order features, 196 Gray Level Dependence Matrix (GLDM), 224 Gray Level Size Zone Matrix (GLSZM), 224 Gray Level Run Length Matrix (GLRLM), 70 Neighboring Gray Tone Difference Matrix (NGTDM), and 336 texture features. And z-score normalization is applied to all features.

### 2.5 Determination of imaging

**Figure 2:**
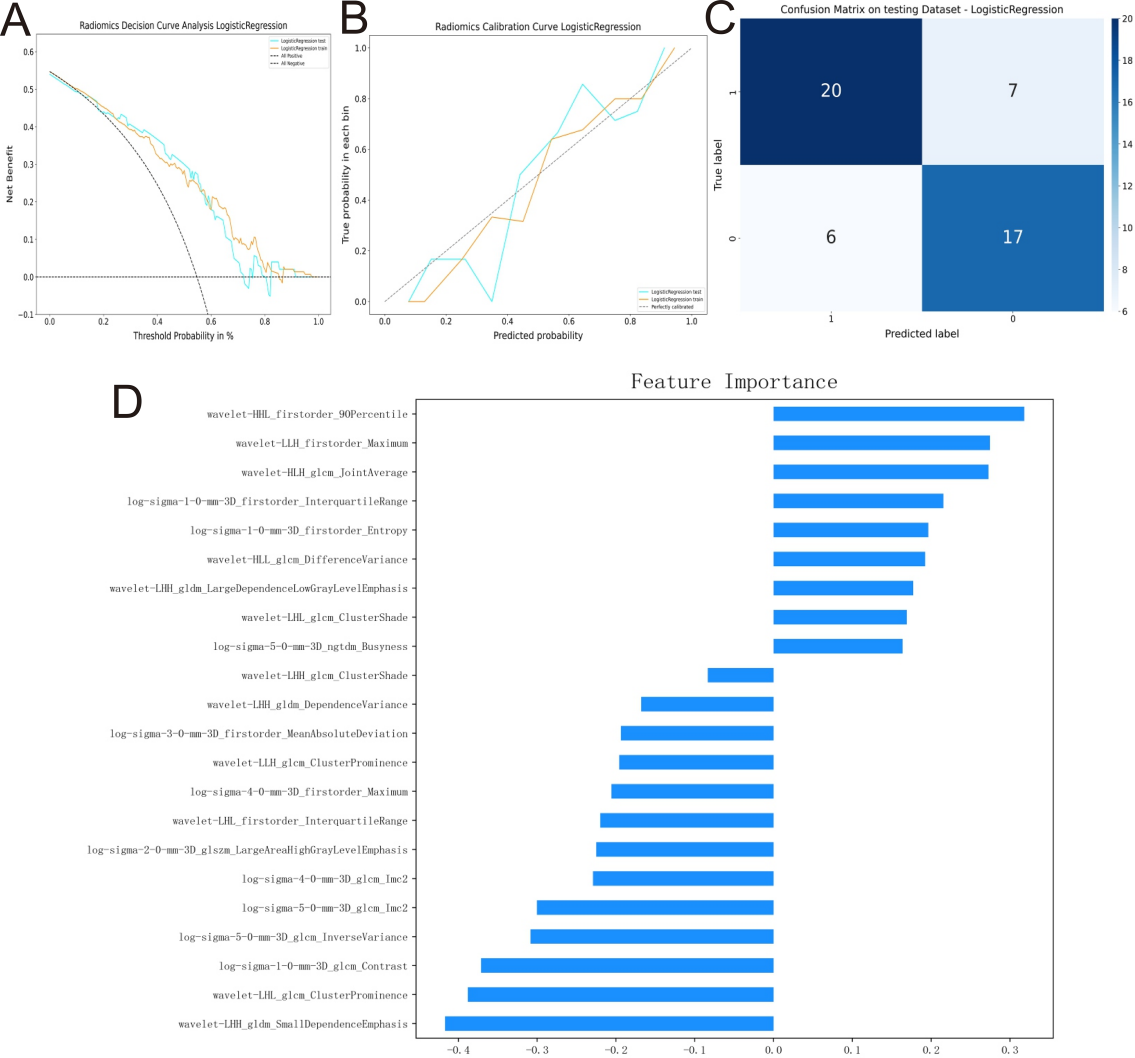
Analysis of LR prediction model efficacy in the validation group. **(A)** is the decision curve of LR in the validation group; **(B)** is the calibration curve of the LR model in the validation group; **(C)** is the confusion matrix of the LR model in the validation group; and **(D)** is the graph of the LR eigen weights.

After standardizing the omics characteristics, we used the “ICC test” software package in R 4.2.1 to calculate the intra-group correlation coefficients for all features. An ICC ≥ 0.75 is considered to indicate good stability, and we screened features with an ICC ≥ 0.75 as predictive features for radiohistology. To reduce the risk of model overfitting, we used the Medical Research Platform (UltraScholar, version 2.0, Numarkun Technology Co., Ltd., https://medresearch.Shukun.net/) to perform K-fold cross-validation on the screened features, setting the folds to 5. We then conducted Pearson correlation analysis to avoid the adverse effects of highly redundant features on model stability and interpretability. Features with absolute correlation coefficients less than 0.8 were selected, and the model complexity was reduced using the Lasso algorithm (Least Absolute Shrinkage and Selection Operator). Based on the above methods, the final predictive features for radiogenomics were determined. After feature selection, the selected features were used to construct the prediction model. In addition to prediction purposes, we visualized the standardized coefficients of each feature in the final prediction model as a measure of its ‘feature importance’ (see **Figure 2D**). This analysis aimed to elucidate the direction of contribution (positive or negative correlation) and relative magnitude of impact of each selected feature on the prediction results, thereby enhancing the model’s interpretability.

### 2.6 Statistical analysis

Using the medical research platform (UltraScholar, version 2.0, Numarkun Technology Co., Ltd., https://medresearch.Shukun.net/) to construct five models, namely Decision Tree, Gaussian Naive Bayes, Logistic Regression, Random Forest, and AdaBoosting, to compare the area under the curve (AUC) of their subjects’ work characteristics, and to select the best model in the comparison. Logistic Regression), Random Forest (Random Forest), AdaBoost (Adaptive Boosting) five models, compare the area under the curve (AUC) of their subjects’ work characteristics to select the best model, and test the effectiveness of the best model in the validation group through the decision curve, the calibration curve, the sensitivity, and the specificity.

## 3 Result

3.1 As shown in **Figure 1**, this study enrolled a total of 198 patients with a mean age of 53.1 years. The baseline data of the patients are summarized in **Table 1**.

**Table 1:** Clinical characteristics of the patients(n=198).

Clinical characteristics	Mean ± SD (range)
Age(years)	53.1 ± 0.6(51.8-54.5)
Size(cm)	2.3 ± 0.1(2.1-2.6)
Histologic grade	
I	4(2)
II	86(43.4)
III	108(54.5)

**Figure 3:**
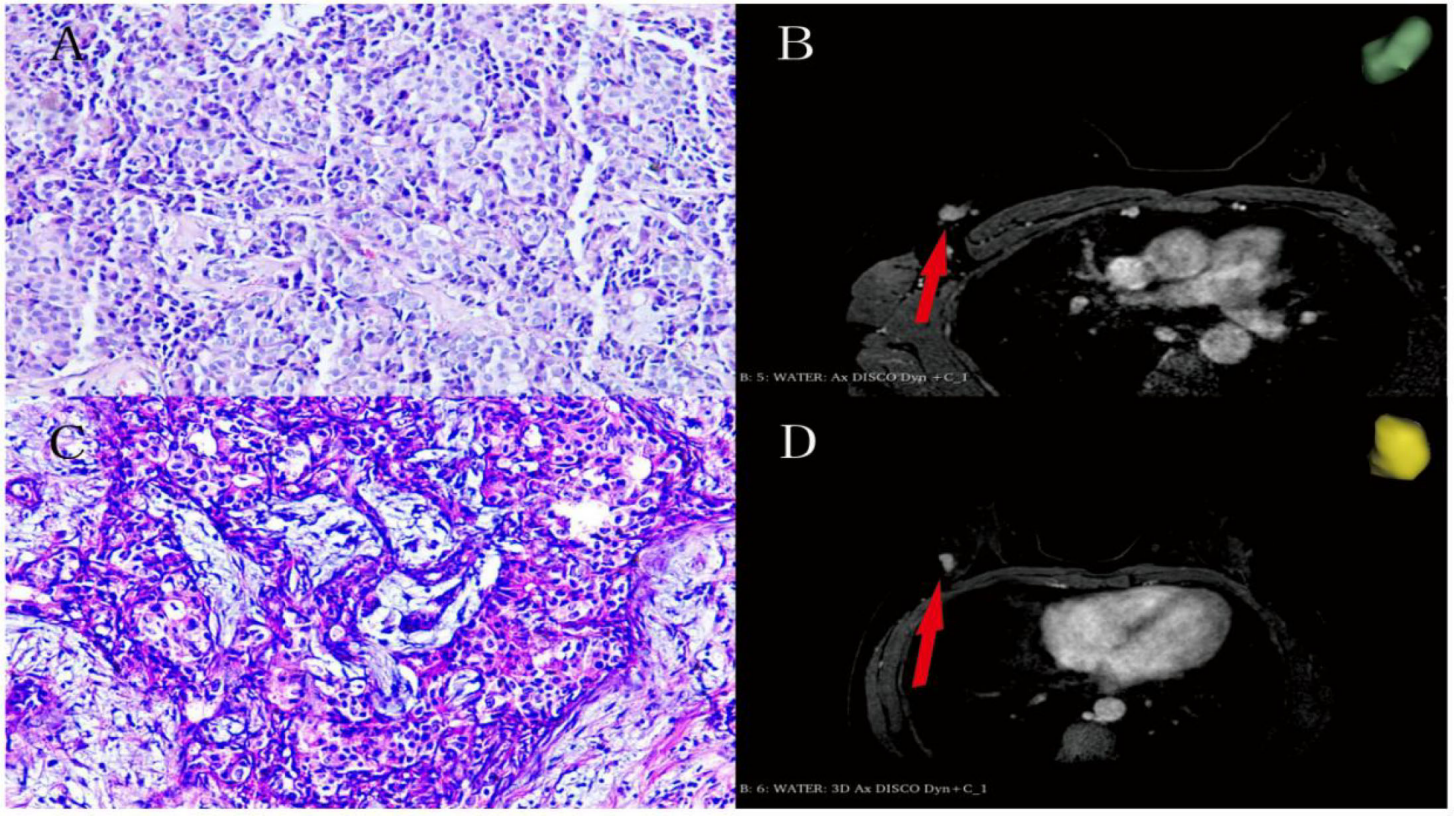
**(A, B)** Female, 48 years old enhanced MRI scan of right breast cancer (red arrow direction) with pathologic grade III (hematoxylin-eosin stain; original magnification, ×100). **(C, D)** Female, 43 years old enhanced MRI scan of right breast cancer (red arrow direction) with pathologic grade II (hematoxylin-eosin stain; original magnification, ×100).

3.2 The feature of Radiomics screening 702 features with ICC<0.75 were removed, and the remaining 614 features were screened using 22 features after performing Pearson correlation analysis and applying the LASSO algorithm to select the optimized features, and the cross-validation diagram is shown in **Figure 3**.

**Figure 4:**
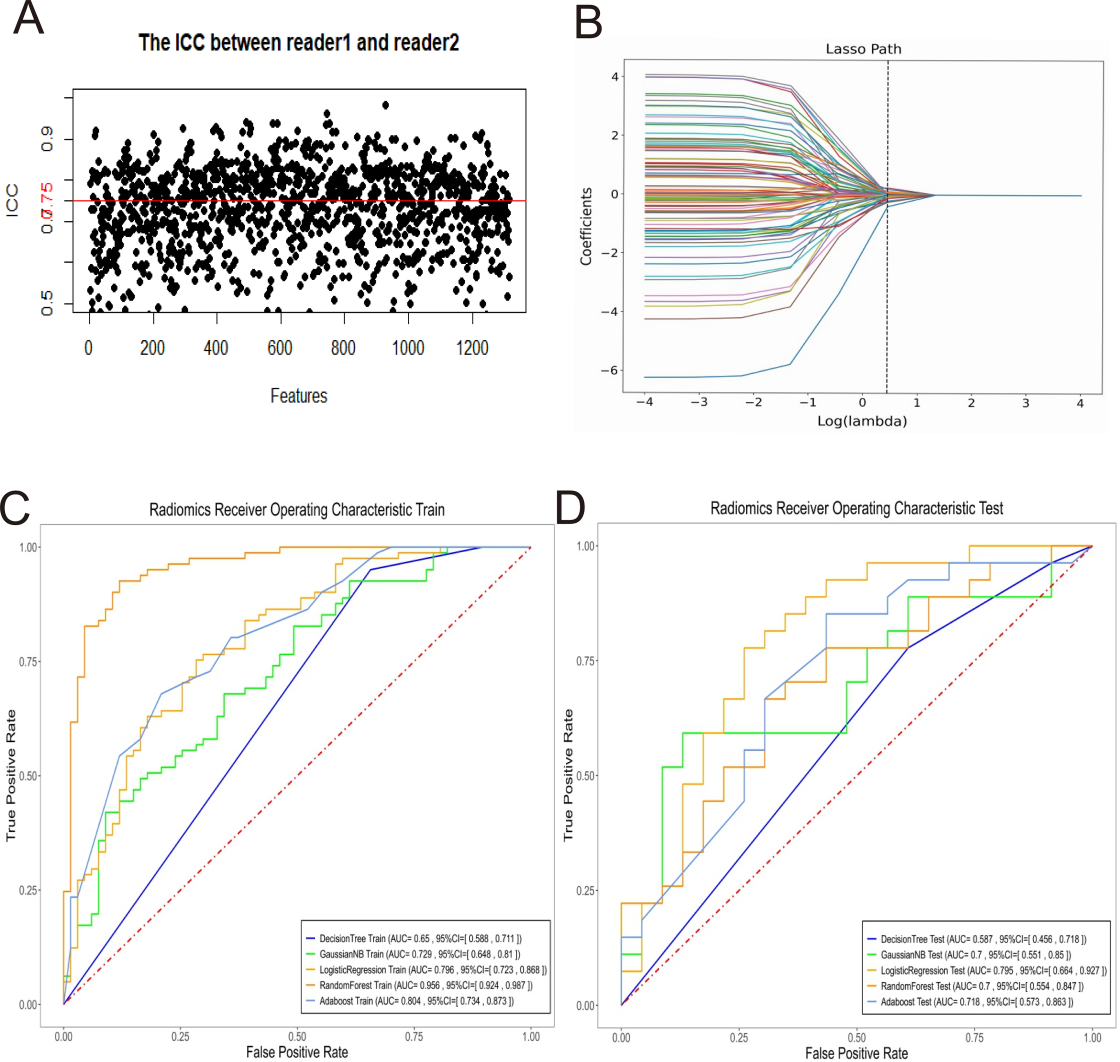
Selection of radiomic features, and construction and validation of predictive models. **(A)** Intragroup correlation coefficients. **(B)** Plot of feature regression coefficients. **(A, B)** Determination of the most significant features to construct the radiomic model. ROC line plots for each model in the training group **(C)** and test group **(D)**.

3.3 Multiple training model establishment Decision Tree, Gaussian Naive Bayes, Logistic Regression, Random Forest, AdaBoost, and Adaptive Boosting are constructed as 5 different models. models, of which the LR prediction model has relatively good stability and is the best prediction model. The performance of each model is shown in **Figure 4**.

3.4 Performance assessment of the best prediction model The LR prediction model has the best prediction effect. The AUC of the prediction model in the validation group was 0.795 (95% CI 0.664~0.927), with a sensitivity of 0.741, a specificity of 0.739, an accuracy of 0.740, an F1-score of 0.755, a positive predictive value of 0.769, a negative predictive value of 0.708, a false-negative rate of 0.259, and a false positive rate of 0.261. The details are as shown in **Table 2**.; the decision curve shows that the model has a good net gain over a wide range of thresholds; the calibration curve is closer to the diagonal of the ideal model (**Figure 2**). The weighting coefficients of the image features are visualized as in **Figure 2D**, and the absolute value of the weighting coefficients of the wavelet-LHH_gldm_SmallDependenceEmphasis feature is the largest, which has the greatest impact on the results of the prediction model.

**Table 2:** Performance evaluation indicators for each classification model.

Model	DT		GNB		LR		RF		AB	
	Train	Test	Train	Test	Train	Test	Train	Test	Train	Test
AUC	0.650	0.587	0.729	0.700	0.796	0.795	0.956	0.700	0.804	0.718
SEN	0.950	0.777	0.679	0.592	0.753	0.741	0.925	0.703	0.679	0.555
SPE	0.343	0.391	0.656	0.826	0.716	0.739	0.880	0.565	0.791	0.739
ACC	0.675	0.600	0.668	0.700	0.736	0.740	0.905	0.640	0.729	0.640
F1	0.762	0.677	0.691	0.680	0.757	0.755	0.914	0.678	0.733	0.625
PPV	0.636	0.600	0.705	0.800	0.762	0.769	0.903	0.655	0.797	0.714
NPV	0.851	0.600	0.628	0.633	0.705	0.708	0.907	0.619	0.670	0.586
FNR	0.049	0.222	0.321	0.407	0.246	0.259	0.074	0.296	0.321	0.444
FPR	0.656	0.608	0.343	0.173	0.283	0.261	0.119	0.434	0.209	0.260

DT, DecisionTree; GNB, GaussianNB; LR, LogisticRegression; RF, RandomForest; AB, Adaboost.

## 4 Discussion

This study successfully constructed and validated a predictive model for Nottingham histological grading of breast invasive ductal carcinoma by integrating DCE-MRI imaging omics with multiple machine learning algorithms. This discovery not only validates the value of imaging omics in non-invasive tumor grading, but also expands the application scenarios of DCE-MRI in precision diagnosis and treatment decision support systems for breast cancer., which is consistent with the results of previous studies (17, 18).

### 4.1 Connections and comparisons with previous studies

Accurate histological grading is essential for personalized treatment of invasive ductal carcinoma of the breast. Although current guidelines have standardized the grading process, practical implementation still involves sampling errors and subjective biases, which affect grading consistency and prognosis assessment. As a non-invasive, radiation-free imaging tool that reflects tumor angiogenesis status, DCE-MRI is widely used in breast cancer diagnosis and evaluation (19–22). Tumor angiogenesis is closely related to the degree of malignancy, and high-grade tumors are usually accompanied by more abundant angiogenesis (14, 23, 24). Therefore, DCE-MRI is an important tool for the diagnosis and prognostic assessment of invasive ductal carcinoma of the breast, as demonstrated in previous studies (25). However, visual assessment of DCE-MRI images alone provides limited information. Imagingomics, by analyzing microscopic features such as grayscale and texture, can quantify tumor heterogeneity and offer a novel approach for non-invasive grading (26, 27).

This study, along with those by Anum et al. (28) and Rong et al. (29) has collectively validated the reliability of the DCE-MRI imagingomics model in predicting key pathological features of breast cancer. However, previous studies have primarily focused on post-neoadjuvant response evaluation and axillary lymph node metastasis prediction. This study, by directly addressing the core biological behavior of tumors—histological grading—provides a non-invasive assessment tool that more directly reflects tumor invasiveness. In terms of model construction methodology, this research did not confine itself to a single algorithm. Instead, through systematic comparison of five machine learning models, it identified the logistic regression (LR) algorithm as the optimal performer for the current task. Unlike the above studies, the performance of imaging histology models with different algorithms varies depending on the specific task, so comparing and selecting the best algorithm for building a model for a specific task is a valuable process (30, 31).

### 4.2 Methodological innovation and advantages

The innovation of this study is mainly reflected in the following three aspects:

In the image sequence selection, we optimized the feature extraction timing by selecting images approximately 90 seconds after contrast agent injection (late arterial phase). At this stage, tumor enhancement is typically most pronounced with maximized contrast against normal glandular tissue. This not only enables radiologists to delineate tumor boundaries more precisely but also provides a higher signal-to-noise ratio data foundation for subsequent radiomics feature extraction.

Secondly, in the analysis of the object, we adhere to the principle of the whole tumor volume analysis, which avoids the loss of heterogeneous information that may be caused by the analysis of a single section or part of the tumor region, and makes the extracted features more representative of the overall biological characteristics of the tumor.

Finally, While ensemble learning algorithms such as Random Forest and AdaBoost have demonstrated outstanding performance in numerous imaging omics studies, this research reveals that logistic regression (LR) models with strong interpretability and high stability outperform other approaches in the specific task of predicting histological grades. For analyzing binary outcomes, logistic regression was able to handle both continuous and categorical predictor variables and allowed adjustment for multiple predictors, thereby effectively reducing potential bias between comparison groups. In contrast to linear regression, logistic regression avoids predictive values beyond 0 and 1 due to the nature of binary outcomes, making it an extremely suitable analytical tool (32). This suggests that in clinical translation-oriented research, the complexity of models should not be prioritized over performance, interpretability, and ease of clinical deployment.

### 4.3 Limitations and future directions

This study, while insightful, is not without limitations.

First, The single-center design is the primary limitation. The single-source sample may introduce selection bias, and the model’s generalization across different medical institutions, MRI scanners, and parameters remains unverified. Future multicenter, prospective cohort studies are required to validate and calibrate the model.

Second, the pathological grading system has been simplified. Due to the limited number of low-grade (G1) cases, this study had to merge intermediate-grade (G1+G2) cases and conduct binary classification with high-grade (G3) cases. While this simplification remains valuable for clinical decision-making (e.g., determining whether more intensive treatment is needed), it compromises the ability to distinguish between G1 and G2 cases, which is crucial for the precise selection of certain treatment strategies. Expanding the sample size and developing a model capable of differentiating the three categories will be a key focus for future research.

Thirdly, the issue of feature stability and standardization. The imaging features are easily affected by factors such as scanning protocol, reconstruction algorithm and segmentation tool. Although the standardization of the process is attempted in this study, the cross-institutional feature stability test has not been carried out.

Fourth, clinical integration of the model. Currently, the model functions as a standalone hierarchical prediction tool. Future research should explore its deep integration with clinical-pathological factors (e.g., age) to develop multimodal prediction line charts or decision support systems, and conduct cost-effectiveness trials to empirically validate its ability to improve patient outcomes.

## 5 Conclusion

In conclusion, we have developed a new model that provides a diagnostic idea for predicting histological grading using intra-tumor imaging features in invasive ductal carcinoma of the breast. This noninvasive preoperative predictive model can be used as a valuable clinical aid in diagnostic modalities. It is believed that with further relevant studies, DCE-MRI imaging histology of the breast will provide more valuable information for predicting histologic grading in patients with invasive ductal carcinoma of the breast.

## Data Availability

The datasets presented in this article are not readily available because <b>Sharing or publicly disclosing the raw data is prohibited. Data access is restricted to authorized personnel only. Copying, distribution, or resale of the original data is not permitted. Original data containing personally identifiable or sensitive information must not be included in any reports or publications. Ensure data is adequately anonymized and protected for privacy and confidentiality when utilized.</b>. Requests to access the datasets should be directed to Chengjun Zhang, zhangchengjun3@163.com.
